# Supplementary material for: The impact of environmental regulation on regional economic growth: A case study of the Yangtze River Economic Belt, China
Source: PLoS One. 2023 Sep 11;18(9):e0290607. doi: 10.1371/journal.pone.0290607 (PMC10495021; doi:10.1371/journal.pone.0290607)
Supplement: Attachment — Submitted filename: Detailed Response to Reviewer.docx [file pone.0290607.s001.docx]

**Responses to reviewers’ comments**

**Dear Editor,**

We greatly appreciate the opportunity to submit a revised manuscript, as well as our response to the reviewer's comments. The manuscript has been carefully revised or provided with valuable and enlightening comments based on the reviewer's comments, including fixing language and formatting errors.

We are providing a clean copy of the revised manuscript along with a version that highlights the changes. The responses to each comment are as follows:

**Reviewers’ comments**

***Reviewer #1:***

1.In the introduction, the theoretical and technical information between is not given. The subject is covered with general statistics and rankings. If variables are added to the model for analysis, their theoretical and technical connections and background should be explained. Otherwise, every desired variable should not be added to the model. In addition, the channels through which these explanatory variables affect the dependent variable should be explained.

**Reply：Thank you for your comment.** **We have added theoretical and technical information about both in the introduction. In Part 3.2, we explain the theoretical and technical connections and backgrounds of the variables, and explain the channels through which these explanatory variables affect the dependent variables**

2. The gap in the literature should be explained in detail and the importance and contribution of the study should be clearly stated.The literature review section is thin with respect to the major study areas, it is required substantial improvement.

**Reply：Thank you for your comment.** **We have made a large number of revisions to the literature review, deleted the literature with repetitive content, not distinctive features or not closely related to the theme, and explained the characteristics, differences and contributions of the literature. The importance of this research is also pointed out**

3. While presenting the empirical results, the study should focus to compare the results obtained and the results of previous studies, and discuss the differences discovered. Are there any other studies in the literature that found similar results? Do the findings overlap with only one study?

**Reply：Thank you for your comment.** **We compared the results of this study with other studies that came to similar conclusions. The differences and their causes are pointed out**

4. Conclusion restates the findings, which is inappropriate. It is recommended that authors use no more than three sentences to sum up the findings. More importantly, the policies are extremely ambiguous and appear to have been predetermined by the authors before the study was even started. The policy recommendations should follow logically from the presentation of the findings and not add anything new.

**Reply：Thank you for your comment.** **We cut out the unnecessary content of the research conclusion, and put forward the suggestion that is closely related to the conclusion of this study**

5. I also think that the study needs proof and editing. I would recommend checking it especially in terms of typo errors.

**Reply：Thank you for your comment.** **We checked the article and corrected the words and punctuation in question firstly**

***Reviewer #2:***

I congratulate the authors for a significant contribution in the field and therefore I recommend the manuscript for publication in its current form. I hope, the authors will continue their efforts to make contribution in their field in future.

**Reply：Thanks very much for your kindly comment.**
